# Intermediate-Term Storage of Spotted Halibut (*Verasper variegatus*) Sperm: Effects of Storage Methods, Extenders Supplemented with Antibiotics and Antioxidants on Sperm Quality

**DOI:** 10.3390/antiox12010122

**Published:** 2023-01-03

**Authors:** Irfan Zidni, Hyo-Bin Lee, Ji-Hye Yoon, Jung-Yeol Park, Hyun-Seok Jang, Youn-Su Co, Dian Yuni Pratiwi, Han-Kyu Lim

**Affiliations:** 1 Department of Biomedicine, Health & Life Convergence Science, BK21 Four, Mokpo National University, Muan 58554, Republic of Korea; 2 Department of Fisheries, The Faculty of Fisheries and Marine Science, Universitas Padjadjaran, Sumedang Regency 45363, Indonesia; 3 Department of Aquaculture and Aquatic Science, Kunsan National University, 558 Daehak-ro, Gunsan 54150, Republic of Korea; 4 Department of Fishery Biology, Pukyong National University, Busan 48512, Republic of Korea; 5 Department of Marine and Fisheries Resources, Mokpo National University, Muan 58554, Republic of Korea

**Keywords:** *Verasper variegatus*, sperm, short-term storage, antioxidants, sperm motility, cell viability

## Abstract

Intermediate-term preservation of sperm assists the reproductive management of fish spermatozoa; however, no information is available on sperm of the spotted halibut, *Verasper variegatus*. We aimed to identify the optimum diluents, temperatures, dilution ratios, antibiotics, and antioxidants for sperm motility and cell viability. The diluents evaluated were marine fish Ringer’s solution (MFRS), Stein’s solution, 300 mM sucrose, and 300 mM glucose (diluted 1:1 [sperm: diluent], 1:2, 1:4, and 1:10 and stored at 0, 2, 4, and 6 °C). Neomycin and gentamycin (100, 200, 400, and 800 mg/L) and antioxidants (Mito-TEMPO [0, 25, 50, 75, 100, 125, 150, 175, and 200 µM], reduced glutathione [0, 2, 4, 6, 8, and 10 mM], and trehalose [0, 50, 100, 150, 200, and 250 mM]) were assessed in terms of sperm preservation. The most effective condition for cold storage of spotted halibut sperm was Stein’s solution at a dilution ratio of 1:4 at 2 °C, with a combination of neomycin 800 mg/L and 250 mM trehalose that showed spermatozoa motility of > 43% after 60 days. These storage conditions will be valuable for spotted halibut hatcheries.

## 1. Introduction

The spotted halibut (*Verasper variegatus*) is categorized is an endangered species in South Korea, Japan, and China [1]. Therefore, a reproductive aquaculture plan is needed to protect this species. Gamete conservation is important for fish reproduction in the aquaculture industry [2]. Short-term storage is often used in gamete conservation because it is simpler, easier, and can be implemented in hatcheries [3]. Such short-term storage enables a variety of genetic enhancement initiatives, such as cryopreservation, artificial insemination, and hybridization [4]. In practice, cold storage of sperm promotes egg production in hatcheries because it is a low-cost and easy means of overcoming the insufficient number of males. The effect of short-term storage of fish sperm has been evaluated in marine fish species such as olive flounder (*Paralichthys olivaceus*) [5], marbled sole (*Limanda yokohamae*), brown sole (*Limanda herzensteini*) [6], starry flounder (*Platichthys stellatus*) [7], yellow croaker (*Larimichthys polyactis*) [8], and giant grouper (*Epinephelus lanceolatus*) [9]. However, to our knowledge, no study has evaluated the influence of short-term preservation of spotted halibut sperm on sperm function.

The successful storage of fish sperm depends on numerous factors, including extenders, dilution ratio, temperature, and antibiotics [5,7]. During storage, sperm motility, fertilization capacity, cell viability, and energy availability for sperm activation must be maintained [10]. The storage temperature has an important impact on the in vitro survivability of fish gametes. However, species have different temperature tolerances. The storage of the sperm of some fish is enhanced with antibiotics and antioxidants. The success of short-term sperm storage is based on motility and cell viability and is hampered by infection or bacterial development in the sperm volume and/or sperm solution [11]. This reduces fertilizing potential, cell quality, and viability, endangering germplasm [12]. Antibiotics—e.g., penicillin, gentamicin, streptomycin, and neomycin—are used in semen cold storage to prevent these issues. However, the type and effective dose vary depending on the fish species [8].

Storage itself and cell metabolism create free radicals, which damage polyunsaturated fatty acids in the cell membrane and disrupt sperm DNA [13]. An antioxidant is a nucleophilic constituent that inhibits the harmful effects of an oxidative cellular environment. Antioxidants preserve the viability of sperm cells during storage by reducing, stopping, or preventing free-radical processes [14]. Antioxidants have been used to alleviate several fish health problems, such as reproduction and freezing fish spermatozoa [15]. Antioxidants have different effects based on their type and concentration [16].

We tested the effects of diluents on spotted-halibut sperm quality during short-term storage. We also evaluated the influence of sperm diluent ratio, sperm storage temperature, antibiotics, and antioxidants on sperm quality.

## 2. Materials and Methods

### 2.1. Broodstock and Collection of Gamates

The research was performed from January to May 2021. In the Marine Seed Hatchery in Yeosu-si, Jeollanam-do, South Korea, 12 mature male spotted-halibut broodstock of average length 36.06 ± 0.28 cm and average weight 373.4 ± 4.4 g were maintained in seawater at a temperature of 10–12.5 °C, salinity of 30.1 ± 1.4 Practical Salinity Units (PSU), dissolved oxygen of 7.4 mg/L, and pH of 7.8. During the experiment, the fish were twice-daily recipients of commercial floatable feed (Merk, Super Plus 7S, Uiryeong, South Korea). For milt collection, the fish were sedated using 50 mg/L 2-phenoxyethanol. Samples of sperm were obtained by stripping and promptly collected using 1.0 mL plastic syringes (without needle). Care was taken to avoid contaminating sperm with water, urine, blood, or feces. The sperm was transferred to 1.5 mL microtubes in an icebox (constant temperature of 4 °C) and immediately transported to the Marine Aquaculture Laboratory of Mokpo National University, South Korea for assessment. Only sperm of motility > 80% by microscopy were used in this study.

### 2.2. Sperm Motility, Duration Motility, and Cell Viability

Computer-assisted sperm analysis was conducted using the CEROS II instrument (Hamilton Thorne, Inc., Beverly, MA, USA) equipped with a Zeiss Axiolab 5 microscope at 10× magnification, including a CM-040GE camera with 0.4-megapixel resolution at 60 frames per second (JAI, Tokyo, Japan). Each sample was diluted in a ratio of 1:200 with artificial seawater (27 g of NaCl, 0.5 g of KCl, 1.2 g of CaCl_2_, 4.6 g of MgCl_2_, and 0.5 g of NaHCO_3_ per liter of distilled water kept at 4 °C), placed into a Leja slide 20 µm (IMV Technologies, France), and tested at 10 °C of room temperature. The motion characteristics investigated were the percentage of moving sperm (motility); path velocity (VAP), quantifies the speed of the sperm head along its average spatial path; curvilinear velocity (VCL), the average speed measured along the actual point to point path taken by the cell; straight-line velocity (VSL), the average velocity of a sperm head along the straight line between its initial and final detected places; percentage straightness (STR), calculated as VSL/VAP × 100; and percentage linearity (LIN), which is the linearity of the curvilinear trajectory as VSL/VCL × 100 [17]. The duration of motility was assessed by measuring the period between spermatozoa activation and their full cessation of activity.

The viability of sperm was evaluated using the Cell Counting Kit-8 (CCK-8) test (Bimake, Houston, TX, USA) as described previously [17]. At a volume ratio of 1:10, CCK-8 reagent was directly applied to the cells in a culture medium. Initially, 100 µL of cell suspension were added to each well of 96-well plates. The cells and reagent (10 µL per well) were incubated for 1–4 h until an orange color formed. Using a microplate reader, the absorbance at 450 nm was determined (Spectra Max 190, Molecular Devices, San Jose, CA, USA). The amount of formazan (orange dye) produced by dehydrogenase activity indicates the number of live cells. White sperm were deemed non-viable while orange sperm were deemed viable.

### 2.3. Experimental Design

#### 2.3.1. Effect of Diluent

To identify the optimum diluent, sperm was diluted 1:2 (sperm: diluent) with Stein’s solution (NaCl 0.75 g, KCL 0.038 g, Hen egg yolk 100 mL, C_6_H_12_O_6_ 0.10 g, and NaHCO_3_ 0.20 g L^−1^ distilled water), marine fish Ringer’s solution (MFRS) (NaCl 13.50 g, KCL 0.60 g, CaCl_2_ 0.35 mL, NaHCO_3_ 0.03, and MgCl_2_ g L^−1^ distilled water), 300 mM sucrose, or 300 mM glucose. The solutions were loaded in 1.5 mL microfuge tubes and refrigerated at 4 °C. Sperm motility, kinematic characteristics, duration motility, and survival were assessed at 5-day intervals until spermatozoa ceased movement.

#### 2.3.2. Effect of Temperature

Sperm samples were diluted 1:2 (sperm: Stein’s solution), loaded in 1.5 mL microfuge tubes, and kept at 0, 2, 4, or 6 °C in a refrigerator. Three replicates were performed. Sperm motility, kinematic characteristics, duration motility, and viability were evaluated at 5-day intervals until spermatozoa ceased movement.

#### 2.3.3. Effect of Dilution Ratio

Sperm were diluted in Stein solution at 1:1, 1:2, 1:4, and 1:10 (sperm: diluent). In triplicate, the solutions were put in 1.5 mL microfuge tubes and refrigerated at 2 °C. Sperm motility, kinematic characteristics, duration motility, and viability were evaluated at 5-day intervals until spermatozoa ceased movement.

#### 2.3.4. Effect of Antibiotics

Sperm were diluted at 1:4 (sperm: Stain’s solution) in triplicate, neomycin and gentamycin (100, 200, 400, and 800 mg/L) were added, and the samples were stored in a refrigerator at 2 °C. Sperm motility, kinematic parameters, duration motility, and viability were evaluated at 5-day intervals until spermatozoa creased movement.

#### 2.3.5. Effect of Antioxidants

Sperm were diluted 1:4 in Stain’s solution with the optimum neomycin or gentamycin concentration (as determined in Section 2.3.4) containing trehalose (0, 50, 100, 150, 200, and 250 mM), GSH (0, 2, 4, 6, 8, and 10 mM), or MT (0, 25, 50, 75, 100, 125, 150, 175, and 200 μM). Three replicates of each treatment were conducted, and samples were refrigerated at 2 °C. Sperm motility and viability were evaluated at 5-day intervals until spermatozoa ceased movement.

#### 2.3.6. Statistical Analysis

The variables are presented as means and standard errors, and the percentage data was normalized prior to analysis via arcsine square root transformation. For the analysis, the SPSS version 23 software was used. One-way ANOVA and Duncan’s multiple-range test (*p* > 0.05) were selected for statistical evaluation. 

## 3. Results

### 3.1. Effect of Diluents

**Figure 1:**
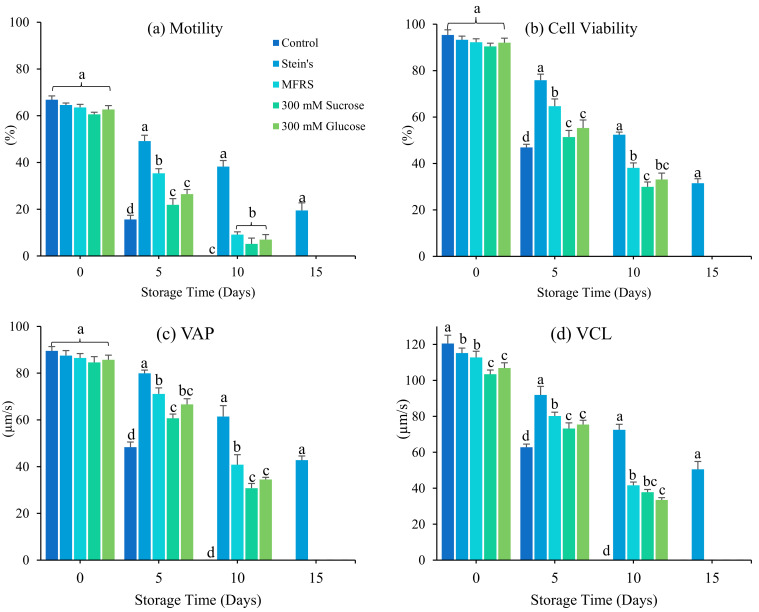

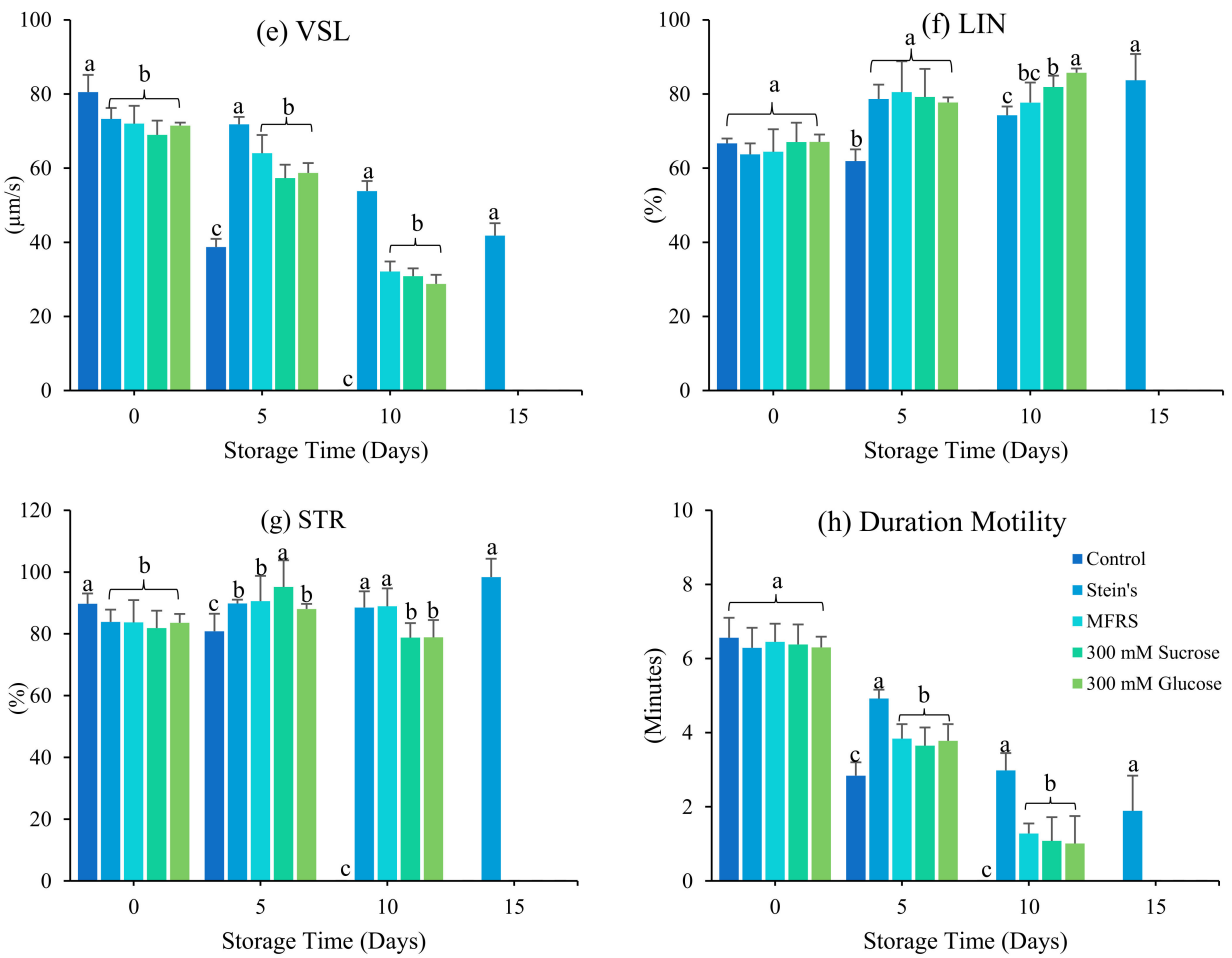
Effects of diluents on sperm of spotted halibut (*Verasper variegatus*) stored for 15 days at 4 °C. (**a**) Motility (%), (**b**) cell viability (%), (**c**) average path velocity (VAP; µm/s), (**d**) curvilinear line velocity (VCL; µm/s), (**e**) straight-line velocity (VSL; µm/s), (**f**) linearity (LIN; %), (**g**) straightness (STR; %) and (**h**) duration motility (minutes). Various lowercase letters represent substantial changes across storage diluents (*p* < 0.05). Control, fresh sperm; MFRS, marine fish Ringer’s solution.

Initial sperm motility and cell viability (day 0) were > 65% in all treatment groups (*p* < 0.05). Diluent significantly affected sperm motility, kinematic parameters, duration motility, and viability during short-term storage (*p* < 0.05). The percentage sperm motility in samples stored without diluent (control) decreased significantly after 5-day storage. Sperm stored in Stein’s solution remained motile for 15 days (19.49 ± 0.31%), whereas sperm stored in MFRS, 300 mM sucrose, and 300 mM glucose remained motile for only 10 days. Sperm velocity and cell viability exhibited similar trends. The optimum storage conditions were Stain’s solution—VAP (42.78 ± 1.79 µm/s), VCL (50.51 ± 4.37 µm/s), VSL (41.80 ± 1.32 µm/s), cell viability (31.53 ± 0.92%), and duration motility (1.89 ± 2.49 min) (Figure 1). Sperm velocity and viability decreased most rapidly in the control group. Stein’s solution was used in subsequent experiments.

### 3.2. Effect of Dilution Ratio

**Figure 2:**
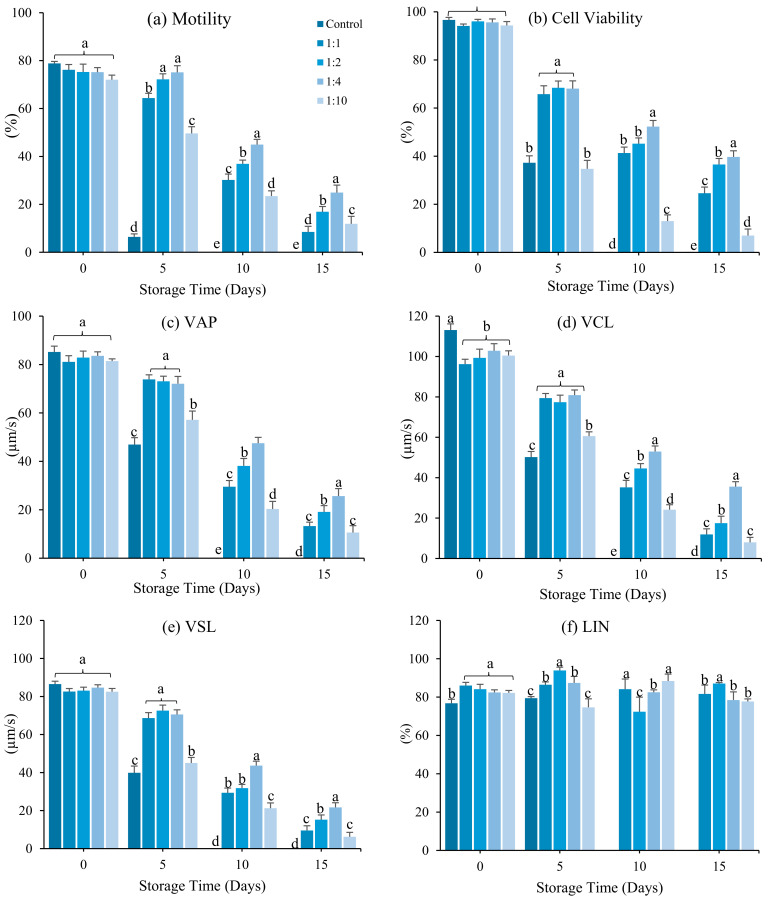

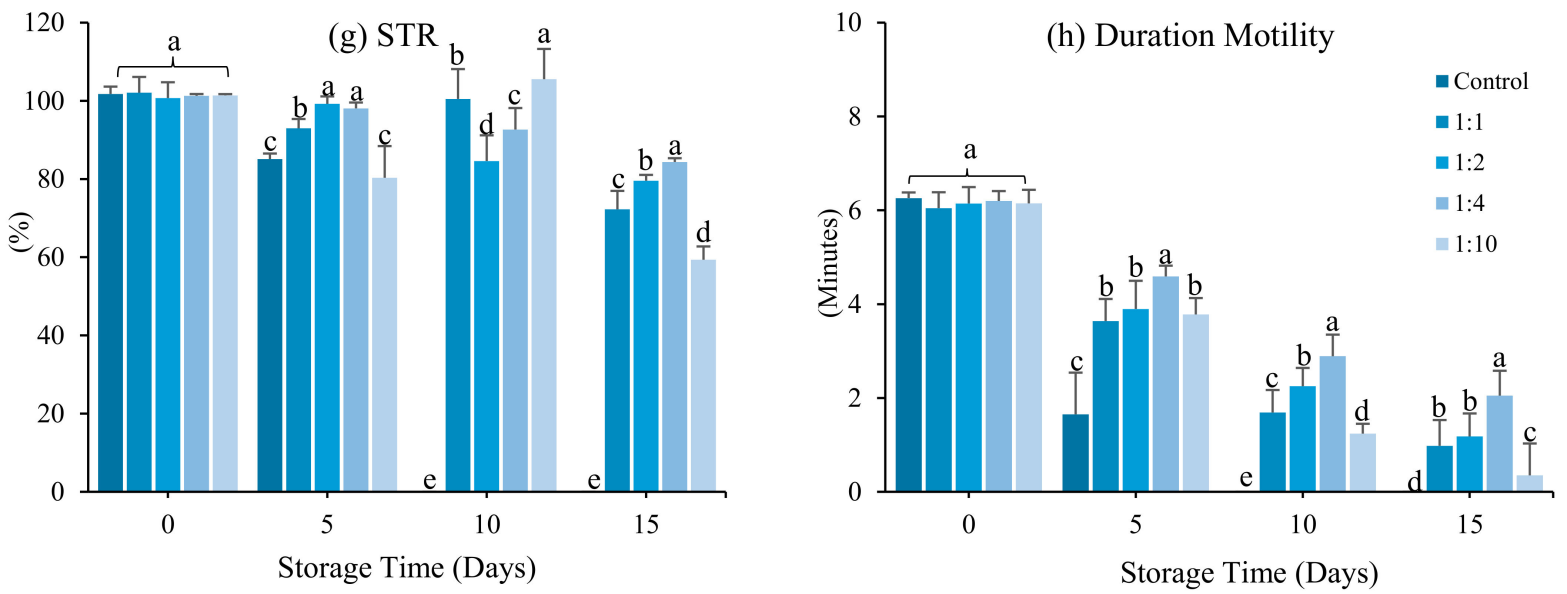
Effect of dilution ratio (sperm: Stein’s solution) on sperm of spotted halibut (*Verasper variegatus*) stored for 15 days at 4 °C. (**a**) Motility (%), (**b**) cell viability (%), (**c**) average path velocity (VAP; µm/s), (**d**) curvilinear line velocity (VCL; µm/s), (**e**) straight-line velocity (VSL; µm/s), (**f**) linearity (LIN; %), (**g**) straightness (STR; %) and (**h**) duration motility (minutes). Various lowercase letters represent substantial changes across dilution ratio (*p* < 0.05). Control, fresh sperm.

Dilution ratio significantly affected sperm motility, kinematic parameters, viability, and duration motility during short-term storage (*p* < 0.05). Sperm storage in Stein’s solution at a 1:4 sperm: diluent ratio significantly increased (*p* < 0.05) sperm motility (24.91 ± 2.33%), VAP (25.67 ± 1.10 µm/s), VCL (35.57 ± 1.31 µm/s), VSL (21.68 ± 1.15 µm/s), and duration motility (2.05 ± 2.05 min) compared to a 1:1, 1:2, or 1:10 ratio (Figure 2) for 15 days. However, cell viability did not differ significantly (*p* < 0.05) at a 1:2 dilution ratio. Therefore, a 1:4 dilution ratio was used in subsequent experiments.

### 3.3. Effect of Storage Temperature

**Figure 3:**
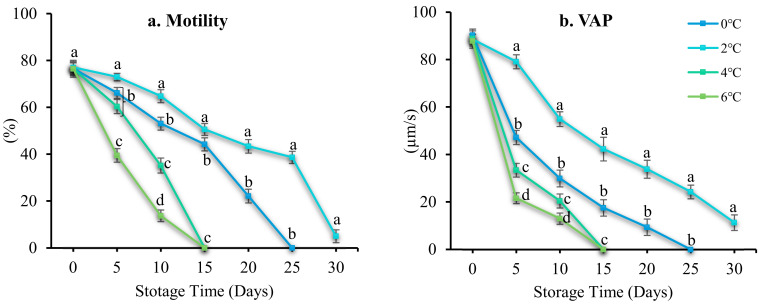

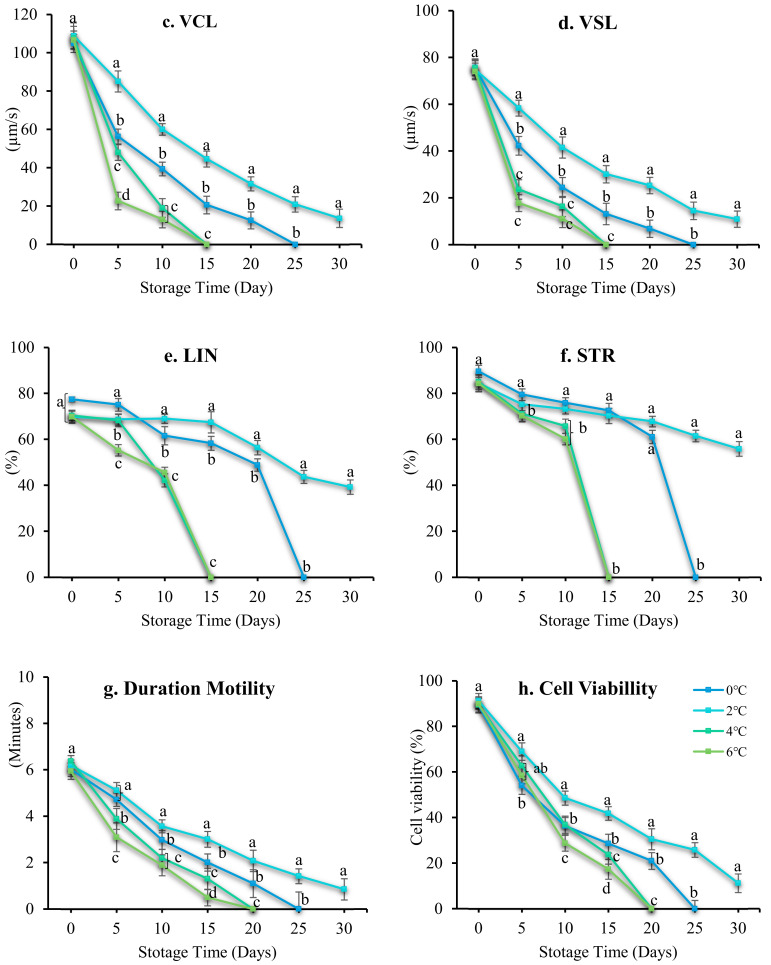
Effect of storage temperature on the function of spotted halibut (*Verasper variegatus*) for 15 days at 4 °C. (**a**) Motility (%), (**b**) average path velocity (VAP; µm/s), (**c**), curvilinear line velocity (VCL; µm/s) (**d**) straight-line velocity (VSL; µm/s), (**e**) linearity (LIN; %), (**f**) straightness (STR; %), (**g**) duration motility (minutes), and (**h**) cell viability (%). Various lowercase letters represent substantial changes across storage temperatures (*p* < 0.05).

Storage temperature significantly affected sperm motility, kinematic parameters, duration motility, and viability during short-term storage (*p* < 0.05). Sperm diluted 1:4 in Stein’s solution stored at 2 °C retained their motility (5.04 ± 0.72%), viability (11.24 ± 0.39%), VAP (11.33 ± 1.28 µm/s), VCL (10.95 ± 1.59 µm/s), VSL (13.68 ± 2.77 µm/s), and duration motility (0.85 ± 1.84 min) for 30 days, and for 25 days at 0 °C (Figure 3). Sperm stored at 4 and 6 °C retained their motility, viability, velocity, and duration motility for 15 days. Therefore, 2 °C was used in subsequent experiments.

### 3.4. Effect of Antibiotics

**Figure 4:**
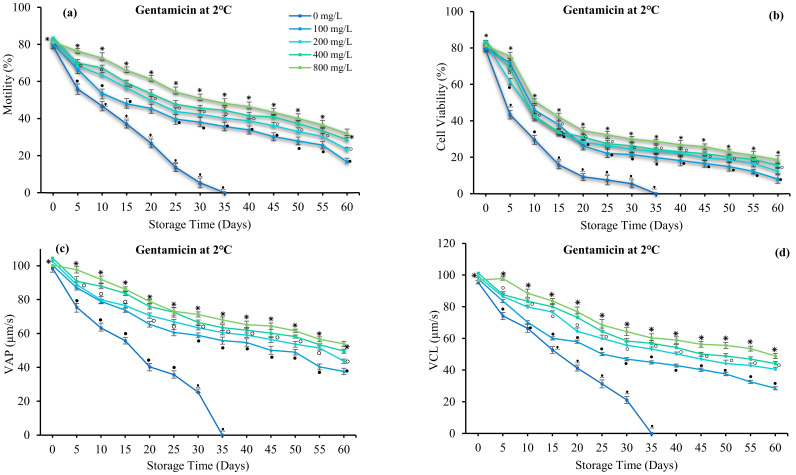

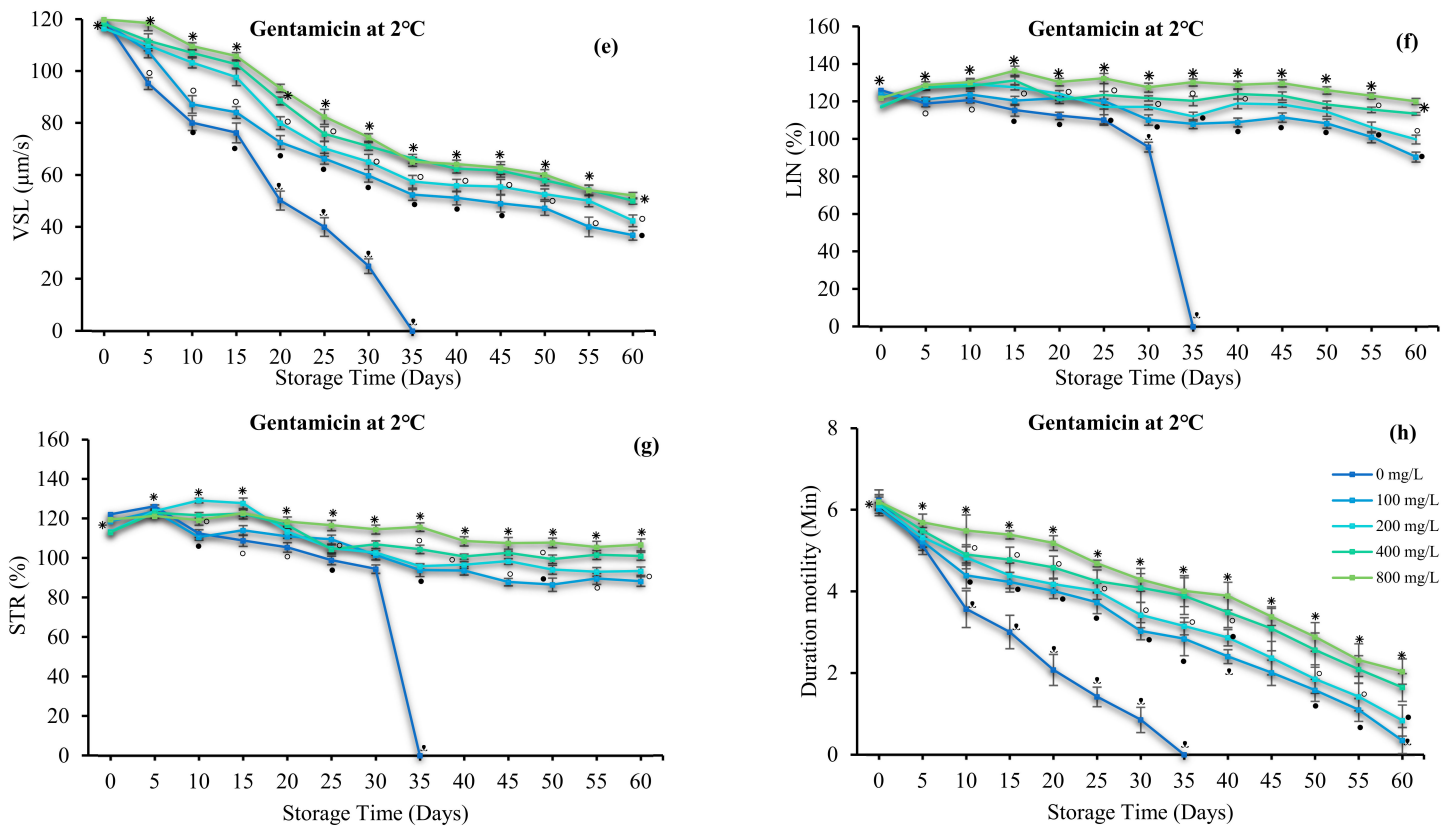
Effect of gentamicin (G) on the sperm of spotted halibut (*Verasper variegatus*) stored in Stein’s solution for 60 days at 2 °C. (**a**) Motility (%), (**b**) viability (%), (**c**) average path velocity (VAP; µm/s), (**d**) curvilinear line velocity (VCL; µm/s), (**e**) straight-line velocity (VSL; µm/s), (**f**) linearity (LIN; %), (**g**) straightness (STR; %), and (**h**) duration motility (minutes). Symbols (⁕,⸰,•,ᵜ) indicate significant differences (*p* < 0.05).

**Figure 5:**
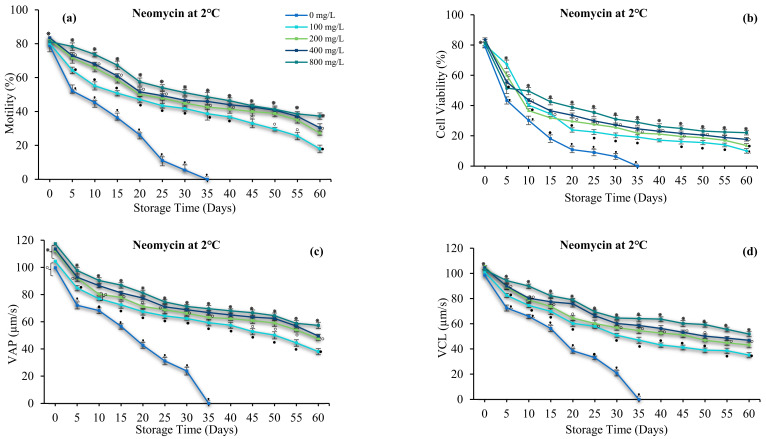

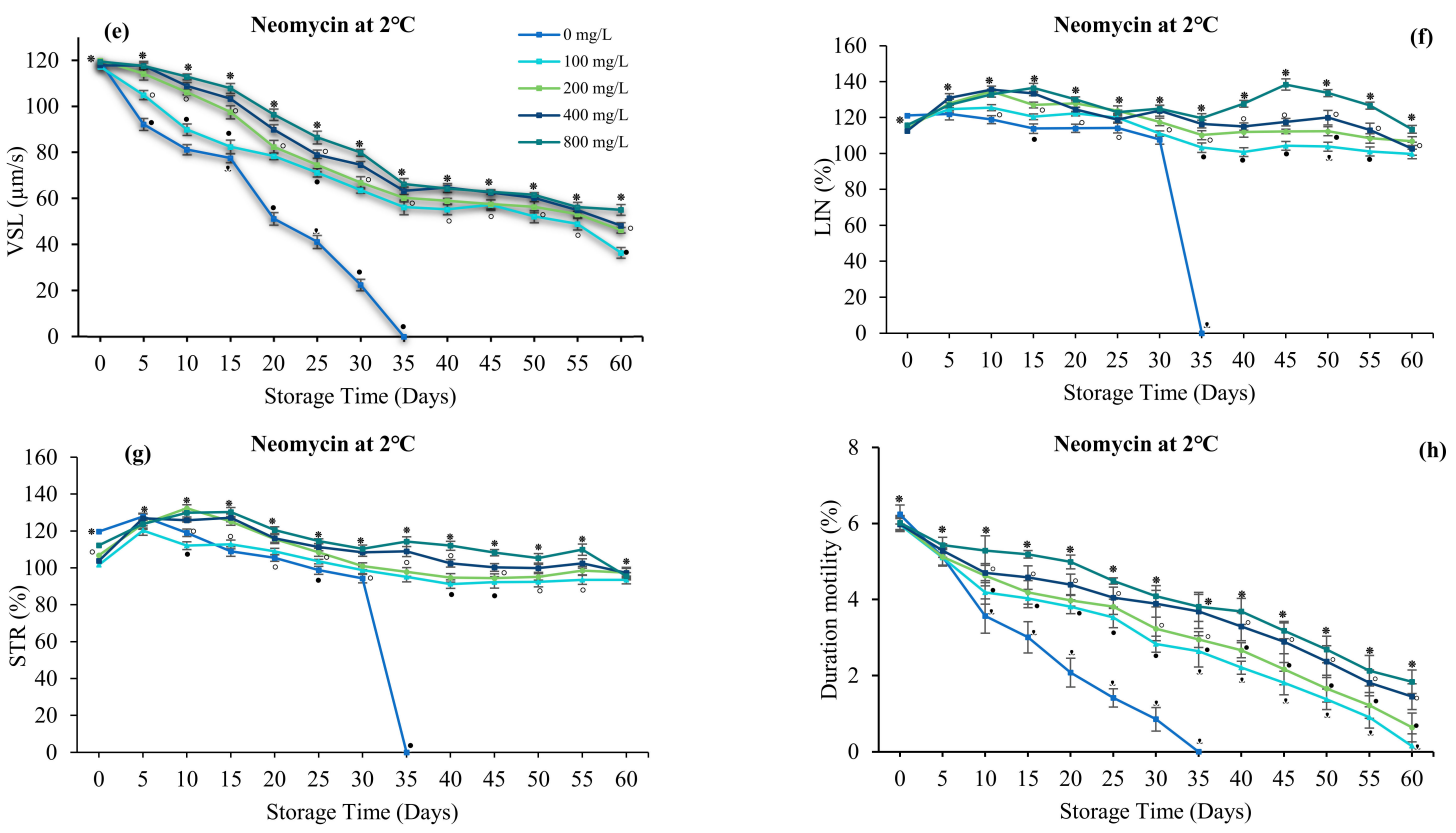
Effect of neomycin (N) on the sperm of spotted halibut (Verasper variegatus) stored in Stein’s solution for 60 days at 2 °C. (**a**) Motility (%), (**b**) viability (%), (**c**) average path velocity (VAP; µm/s), (**d**) curvilinear line velocity (VCL; µm/s), (**e**) straight-line velocity (VSL; µm/s), (**f**) linearity (LIN; %), (**g**) straightness (STR; %), and (**h**) duration motility (minutes). Symbols (⁕,⸰,•,ᵜ) indicate significant differences (*p* < 0.05).3.5. Effect of Antioxidants.

Antibiotics significantly affected sperm motility, velocity, and viability (*p* > 0.05). Sperm quality increased with increasing gentamicin and neomycin concentrations. The use of antibiotics prolonged sperm viability by up to 60 days. In the antibiotic-free group, sperm motility was lower and immotile on day 35. Gentamicin and neomycin at 800 mg/L increased sperm motility (31.92 ± 2.23 and 37.36 ± 2.91%), VAP (53.86 ± 2.70 and 57.43 ± 1.90 µm/s), VSL (52.14 ± 2.39 and 55.10 ± 2.74 µm/s), VCL (50.60 ± 2.71 and 51.94 ± 3.37 µm/s), and viability (18.64 ± 1.46 and 19.13 ± 2.05%) for 60 days in cold storage (Figure 4 and Figure 5). Therefore, 800 mg/L gentamicin and neomycin were used in subsequent experiments.

#### 3.4.1. Mito-TEMPO

**Figure 6:**
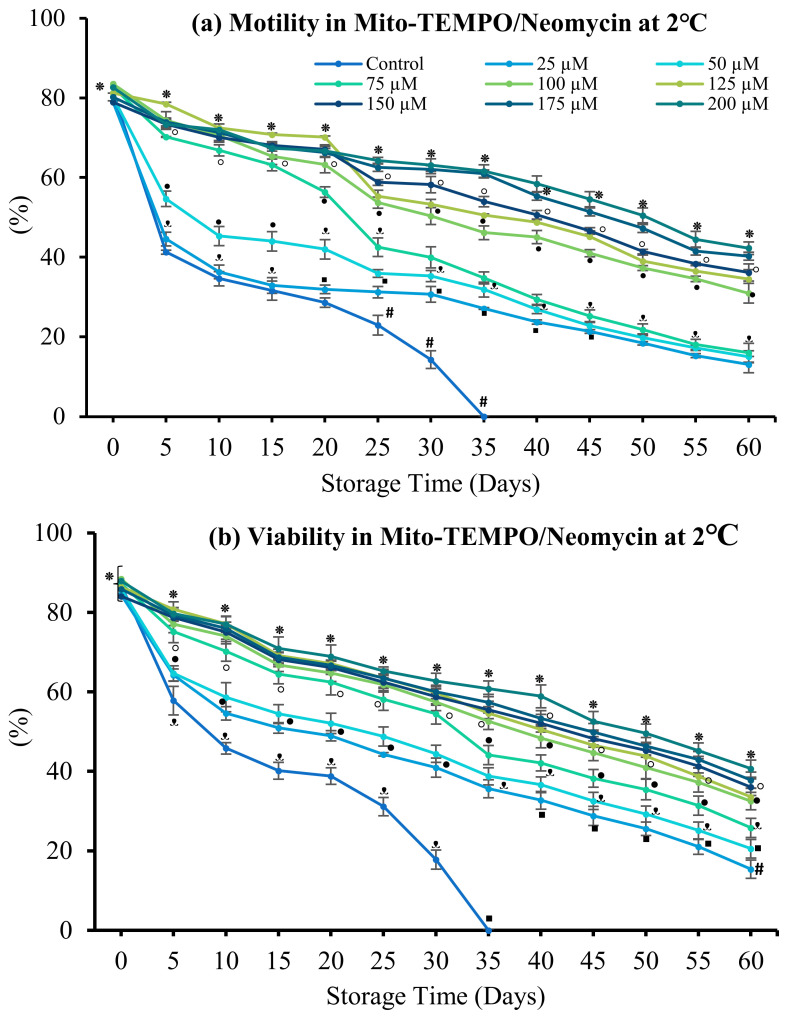

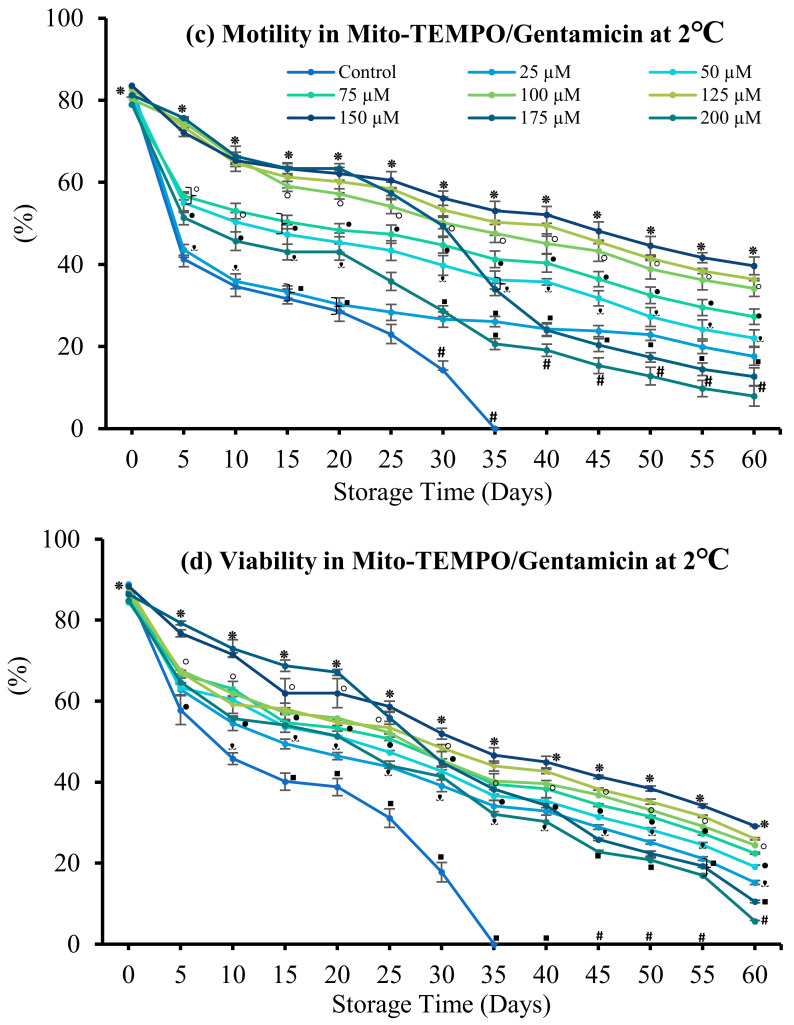
Effect of Mito-TEMPO on spotted halibut (*Verasper variegatus*) sperm stored in Stein’s solution (1:4) + neomycin (N) or gentamicin (G) at 2 °C for 60 days. (**a**) Motility in neomycin (%), (**b**) viability in neomycin (%), (**c**) Motility in gentamicin (%), and (**d**) viability in gentamicin (%). Symbols (⁕,⸰,•,ᵜ,▪,#) indicate significant differences (*p* < 0.05). Control, without antibiotic and antioxidant.

The effects of the indicated concentrations of Mito-TEMPO (MT) in combination with antibiotics (800 mg/L gentamicin or neomycin) on sperm motility and viability are shown in Figure 6. Sperm quality was dose-dependently increased by MT in combination with neomycin. By contrast, sperm quality decreased with increasing MT concentration in combination with gentamicin. Sperm motility and cell viability were maximally increased by 200 µM MT in combination with neomycin (42.30 ± 2.94% and 40.69 ± 3.09%, respectively), followed by 175 µM MT (40.30 ± 1.79% and 37.80 ± 2.38%; *p* > 0.05). The lowest sperm quality resulted from 25 µM MT in combination with neomycin. In the presence of 800 mg/L gentamicin, maximum sperm motility and viability were obtained using 150 µM MT (39.65 ± 1.92% and 29.20 ± 1.88%, respectively), followed by 125 µM MT (36.37 ± 2.40% and 26.05 ± 2.23%) (*p* > 0.05). The lowest sperm quality resulted from 200 µM MT in combination with gentamicin.

#### 3.4.2. Trehalose

**Figure 7:**
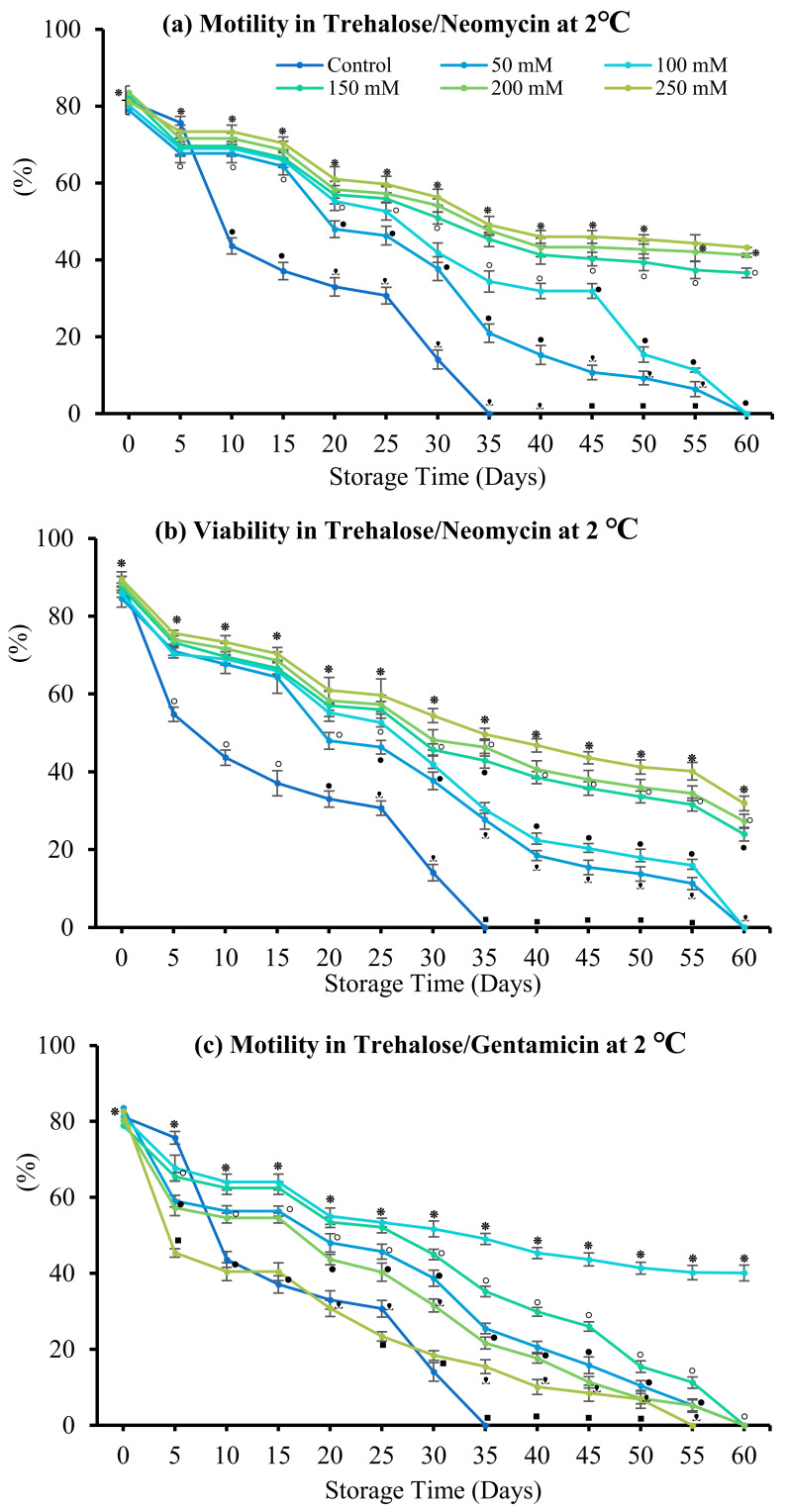

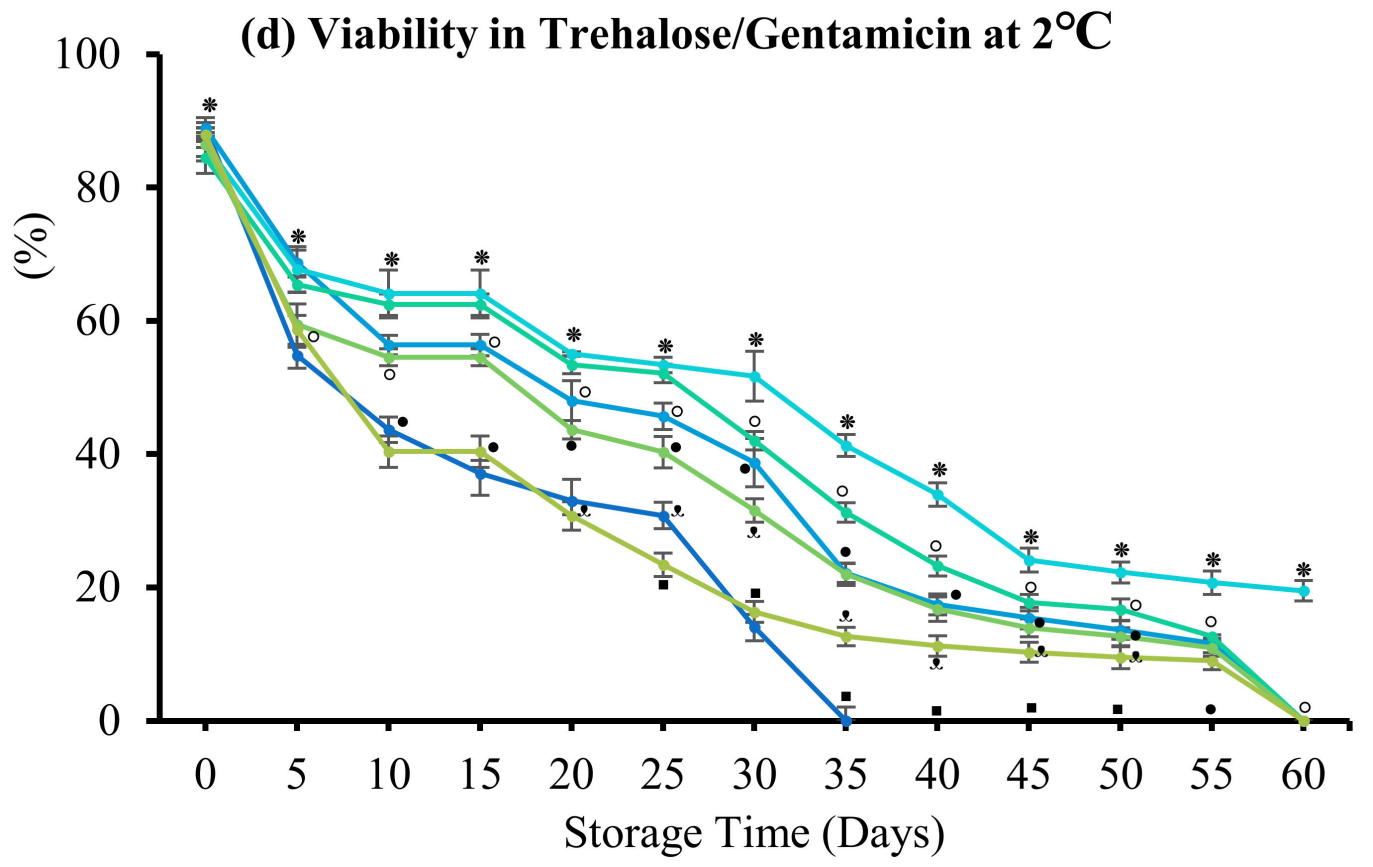
Effect of trehalose on spotted halibut (*Verasper variegatus*) sperm stored in Stein’s solution (1:4) + neomycin (N) or gentamicin (G) at 2 °C for 60 days. (**a**) Motility in neomycin (%), (**b**) viability in neomycin, (**c**) Motility in gentamicin (%), and (**d**) Viability in gentamicin. Symbols (⁕,⸰,•,ᵜ,▪) indicate significant differences (*p* < 0.05). Control, without antibiotic and antioxidant.

During storage for 60 days, 250 mM trehalose + neomycin yielded the highest sperm motility and viability (43.25 ± 1.65% and 31.92 ± 1.93%, respectively), followed by 200 mM trehalose (41.24 ± 2.48% and 27.32 ± 2.17%) (*p* > 0.05) (Figure 7). In the presence of neomycin, sperm quality increased with increasing trehalose concentration. By contrast, in the presence of gentamicin, 100 mM trehalose resulted in the highest sperm motility and viability (40.10 ± 2.18% and 19.50 ± 1.71%, respectively). Sperm motility and viability decreased during storage for 60 days with increasing concentrations of trehalose in combination with gentamicin.

#### 3.4.3. Reduced Glutathione

**Figure 8:**
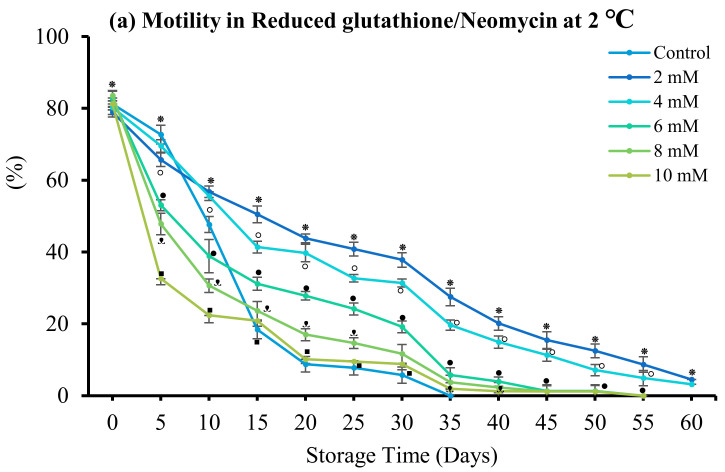

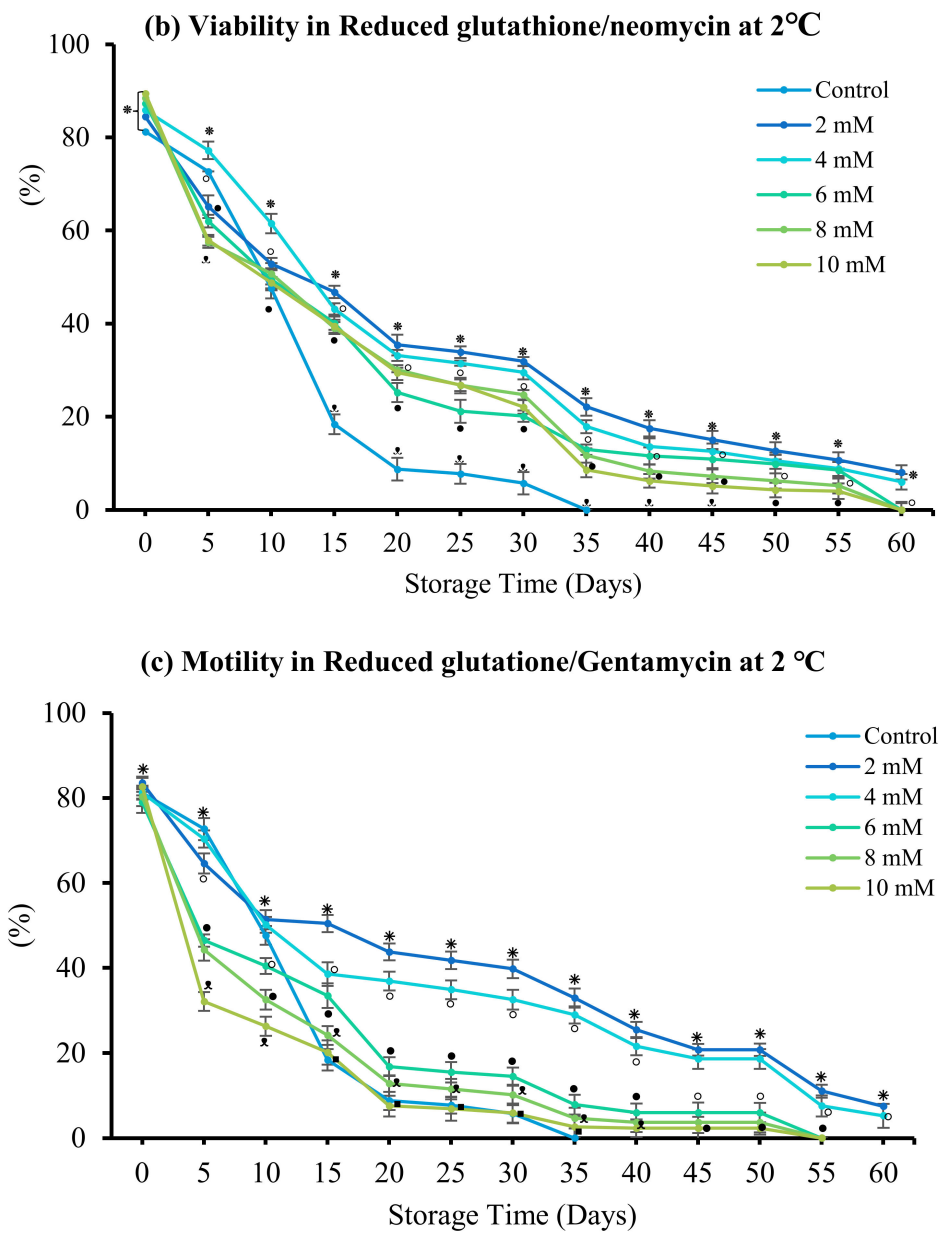

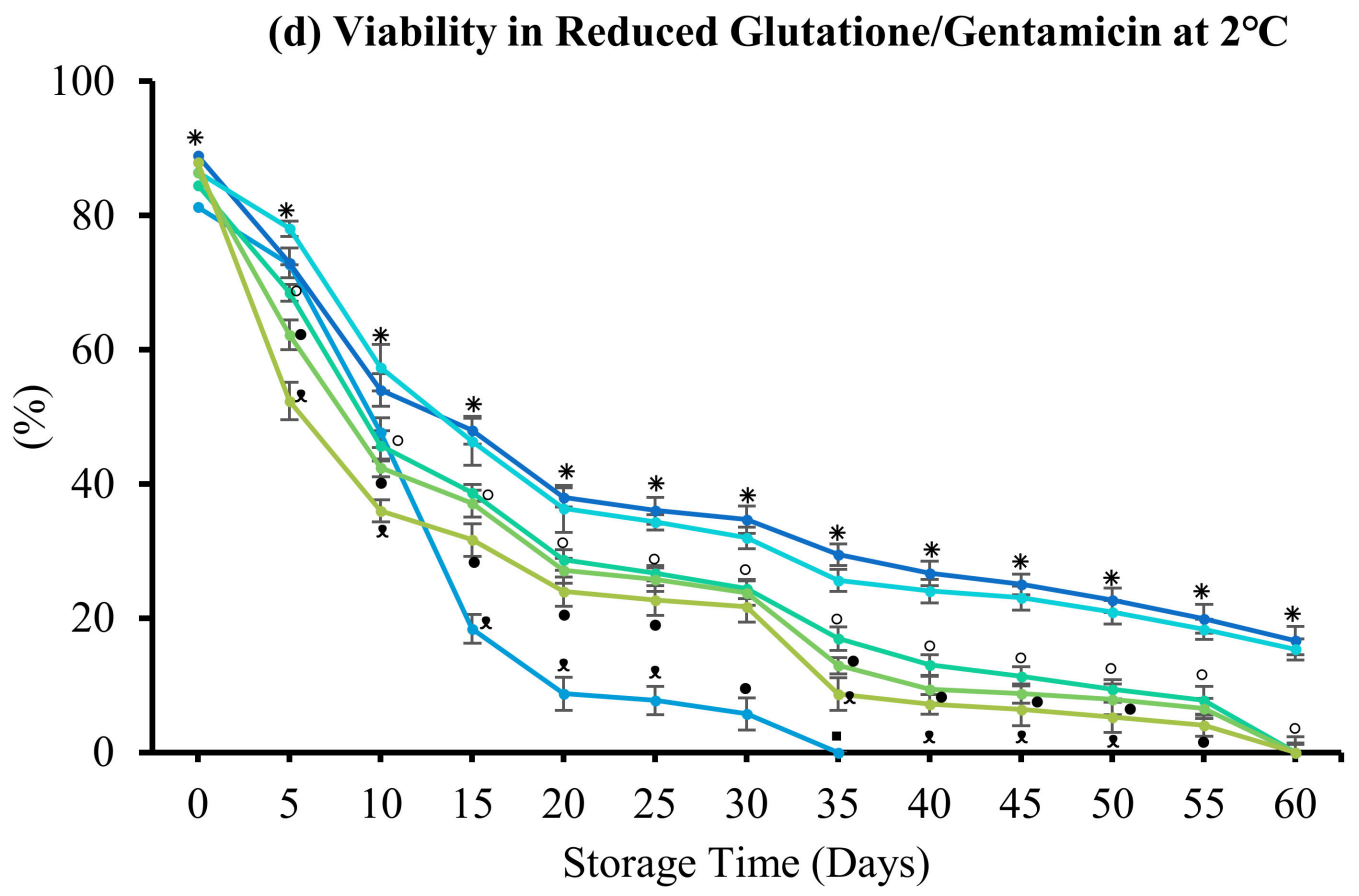
Effect of reduced glutathione (RG) on spotted halibut (*Verasper variegatus*) sperm stored in Stein’s solution (1:4) + neomycin (N) or gentamicin (G) at 2 °C for 60 days. (**a**) Motility in neomycin (%), (**b**) viability in neomycin, (**c**) Motility in gentamicin, and (**d**) Viability in gentamicin. Symbols (⁕,⸰,•,ᵜ,▪) indicate significant differences (*p* < 0.05). Control, without antibiotic and antioxidant.

Sperm motility and viability were maintained during 60 days of storage with 2 mM RG (7.49 ± 2.11% and 16.68 ± 1.92%, respectively) and 4 mM RG (5.25 ± 2.31% and 15.39 ± 2.69%, respectively) in combination with gentamicin. Similarly, 2 mM RG (4.50 ± 2.11% and 8.08 ± 2.77%, respectively) and 4 mM RG (3.20 ± 2.32% and 6.04 ± 2.49%, respectively) combined with neomycin enhanced sperm motility and viability over 60 days (Figure 8). In this treatment group, the quality of stored sperm decreased after 35 days of storage.

## 4. Discussion

Short-term storage of sperm at lower temperatures may promote brood stock upgradation at distantly located hatcheries. This is the first study to develop a storage protocol for spotted halibut sperm. The implementation of sperm short-term storage methods for endangered or threatened fish is an effective conservation strategy for these species. In addition, the short storage of sperm can support artificial fertilization in fish. Sperm motility, kinematic parameters, and viability are important aspects of sperm quality, particularly if the fertilization rate cannot be evaluate because of a lack of mature eggs [7,18]. 

In sperm storage, the extender, which has a composition similar to seminal plasma, maintains sperm function and extends lifespan [19]. The selection of an appropriate extender is crucial for the successful cold storage of fish sperm [7]. In this study, Stein’s solution, was the most effective extender for the cold storage of spotted halibut sperm. Indeed, similar findings have been reported for olive flounder (*Paralichthys olivaceus*) [5], brown sole (*Pleuronectes herzensteini)* [6], marbled sole (*Limanda yokohamae*), and starry flounder (*Platichthyes stellatus*) [7]. In this study, MFRS, 300 mM sucrose, and 300 mM glucose reduced sperm quality. However, MFRS was effective for the short-term storage of the sperm of tiger puffer (*Takifugu rubripes*) [20], yellowtail tetra (*Astyanax altiparanae*) [21], yellow drum (*Nibea albiflora*) [22], and Scapharca broughtonii [22]. A sucrose base increased the quality of the sperm of effective rainbow smelt (*Osmerus mordax*) [23] and glucose that of meagre (*Argyrosomus regius*) [24]. Extender effectiveness differs by fish species. Fish sperm diluents are dependent on the chemical composition of seminal plasma because sperm must be kept stationary to avoid energy depletion [25]. The extender solution should also provide the nutrients and energy sperm needed to survive during storage. Stein’s solution contains NaCl, KCl, egg yolk, NaHCO_3_, and C_6_H_12_O_6_, which exert a buffering effect, tonicity, prevent sperm activation, and protect the membrane, respectively, during cold storage [26]. The medium, dilution ratio (sperm: medium), storage temperature, and antibiotics and antioxidants are determinants of sperm quality after storage.

The dilution ratio should be selected according to fish species to optimize the initial sperm density and the buffer capacity of the diluent. Some species’ spermatozoa are extremely sensitive to dilution [25]. In this study, a 1:4 (sperm: extender) dilution ratio was superior to 1:1, 1:2, and 1:10 ratios in terms of sustaining motility. Similar results have been reported for meagre (*Argyrosomus regius*) [24], tambaqui (*Colossoma macropopum*) [27], walking catfish (*Clarias macrocephalus*) [28], and starry flounder (*Platichthys stellatus*) [7]. The optimum dilution ratio for maintaining motility differs according to fish species. For the Patagonian blenny (*Eleginops maclovinus*) [29], paddlefish (*Polyodon spathula*) [30], and the Mozambique tilapia (*Oreochromis mossambicus*) [31], a 1:1 dilution ratio was optimum. By contrast, a 1:2 dilution ratio was optimum for striped bass (*Morone saxatilis*) [32], yellow drum (*Nibea albiflora*) [22], Atlantic salmon (*Salmon salar*) [10,33], rainbow trout (*Oncorhynchus mykiss*) [34], salmon (*Oncorhynchus mykiss*) [35], trout (*Oncorhynchus mykiss*) [36], and salmonids [10]. A dilution ratio of 1:10 was optimum for yellowtail tetra [21], orangefin labeo (*Labeo calbasu*) [37], perch (*Perca fluviatilis* L.) [38], and Salmonidae milt [39]. For storage, the typical sperm: diluent ratio is 1:1 to 1:20, with the best results at ratios of ~1:3 [2]. In this study, undiluted sperm became immotile by day 10, indicating an insufficient energy resource in the sperm cell. This is presumably because undiluted sperm does not get external nutritional intake such as ionic composition (sodium chloride and potassium chloride) and glucose as other potential sources of energy from diluent, which indicates a relationship to the sperm motility and cell viability. According to Christen et al. 1987 [40], the complete arrest of sperm motility seems to be due to different causes depending upon the ionic composition of the dilution medium. There is evidence that using glucose in a diluent can also maintain sperm motility in viviparous fish [41].

Low temperatures (approaching 0 °C) are typically advised because they decrease the metabolic rate and energy consumption of cells without altering their structural integrity [42,43]. However, for practical reasons, most studies have used 4 °C, the typical temperature of domestic refrigerators. In this study, cold-stored spotted halibut sperm remained motile for 30 days at 2 °C, 20 days at 0 °C, and 10 days at 4 and 6 °C. The most optimum cold storage temperatures were 2 °C for starry flounder (*Platichthys stellatusi*) [7] and 2.5 °C for yellowtail tetra (*Astyanax altiparanae*) [21]. Due to the cold shock resistance of aquatic species sperm, the optimal temperature for sperm storage is 0–4 °C. Low temperatures reduce sperm metabolism and prevent bacterial growth [21,25]. Nile tilapia and channel catfish (*Ictalurus punctatus)* sperm should be stored at 0–4 °C [44,45].

Antibiotics—typically penicillin, erythromycin, gentamicin, streptomycin, and neomycin—are added to prevent bacterial contamination of the stored sperm [46,47]. In this study, sperm motility, velocity, and viability were maintained until day 60 compared to day 30 in the antibiotic-free group. Gentamicin and neomycin at 800 mg/L increased sperm motility, velocity, and viability. The optimum antibiotic concentration varies among fish species. The application of 600 mg/L gentamicin and 200 mg/L neomycin increased the sperm quality of yellow croaker (*Larimichthys polyactis*) [18] and 500 and 750 µg/mL gentamicin for Nile tilapia [12]. In the cold storage of Caspian brown trout (*Salmo trutta caspius*) sperm, penicillin and streptomycin at 500 IU/mL increased sperm motility and viability [48]. Similarly, during sperm storage of the common frog (*Rana temporaria*), sperm quality increased with increasing gentamicin concentration [49]. In Atlantic salmon, antibiotics enhanced fertilization capacity [50]. According to Jenkins & Tiersch [51], cold storage decreased the quality of channel catfish (*Ictalurus punctatus*) sperm because of contamination by a Pseudomonas sp. Therefore, antibiotics may enhance stored sperm quality by suppressing microbial growth and/or activity.

During the cold storage of sperm, antioxidants inhibit free-radical reactions. In this study, 250 mM trehalose with neomycin increased the motility and viability of spotted halibut sperm during storage for 60 days. Indeed, higher concentrations of trehalose further increased the sperm quality. By contrast, in the presence of gentamicin, a high trehalose concentration reduced sperm motility and viability. Trehalose was useful for the cryopreservation of rainbow trout [52], whitefish [53], seabream (*Pagrus major*) [54], and Amazonian catfish (*Leiarius marmoratus*) [55] sperm. Trehalose reduces intracellular ice crystal formation and cryoinjuries caused by freezing by boosting the level of GSH and decreasing lipid peroxidation [56]. In addition, 200 µM MT + neomycin increased sperm motility and viability in a dose-dependent manner. In combination with gentamicin, a high concentration of MT reduced sperm motility and viability. MT at 100 µM was optimal for cryopreservation of spotted halibut sperm [14]. MT reportedly enhances the cryopreservation of testicular tissue of several mammalian species. In addition, MT reduces DNA damage and oxidative stress in variety of cell types [57]. MT maintains sperm function and energy homeostasis via oxidative phosphorylation and ATP synthesis [58]. In rooster semen storage, MT at 5 and 50 µM preserved sperm quality [59]. In this study, 2 and 4 mM RG in the presence of gentamicin or neomycin prolonged sperm viability and motility until 60 days. However, higher concentrations of RG decreased sperm quality. RG participates in antioxidant activity as a substrate for glutathione peroxidase [60]. RG at 2, 4, and 6 mM enhanced the cryopreservation of tambaqui (*Colossoma macropomum*) sperm [61]. RG is also used in the preservation of mammalian sperm. The fertility of cryopreserved rooster sperm was increased by 2 and 4 mM RG [62]. The application of 2 mM RG enhanced boar sperm cryopreservation [63], and 2 and 5 mM RG promoted cryopreservation of ram spermatozoa [64]. RG at 10 mM prevented DNA damage in cryopreserved dog sperm [65].

## 5. Conclusions

Optimal conditions for the storage of spotted halibut sperm were Stein’s solution (extender), a 1:4 sperm: extender dilution ratio, 800 mg/L neomycin, 250 mM trehalose, and a temperature of 2 °C.

## Data Availability

All of the data is contained within the article.
